# Body Composition Assessment by Dual-Energy X-Ray Absorptiometry: A Useful Tool for the Diagnosis of Lipedema

**DOI:** 10.1159/000527138

**Published:** 2022-10-28

**Authors:** Giacomo Buso, Lucie Favre, Nathalie Vionnet, Elena Gonzalez-Rodriguez, Didier Hans, Jardena Jacqueline Puder, Céline Dubath, Chin-Bin Eap, Wassim Raffoul, Tinh-Hai Collet, Lucia Mazzolai

**Affiliations:** ^a^Angiology Division, Heart and Vessels Department, Lausanne University Hospital, University of Lausanne, Lausanne, Switzerland; ^b^Endocrinology, Diabetology and Metabolism Division, Medicine Department, Lausanne University Hospital, University of Lausanne, Lausanne, Switzerland; ^c^Locomotor System Department, Interdisciplinary Centre for Bone Diseases, Lausanne University Hospital, University of Lausanne, Lausanne, Switzerland; ^d^Obstetric Service, Department Woman-Mother-Child, Lausanne University Hospital, University of Lausanne, Lausanne, Switzerland; ^e^Department of Psychiatry, Unit of Pharmacogenetics and Clinical Psychopharmacology, Center for Psychiatric Neuroscience, Lausanne University Hospital, University of Lausanne, Prilly, Switzerland; ^f^Center for Research and Innovation in Clinical Pharmaceutical Sciences, University of Lausanne, Lausanne, Switzerland; ^g^Institute of Pharmaceutical Sciences of Western Switzerland, University of Geneva, University of Lausanne, Lausanne, Switzerland; ^h^Plastic Surgery Division, Locomotor System Department, Lausanne University Hospital, University of Lausanne, Lausanne, Switzerland; ^i^Service of Endocrinology, Diabetology, Nutrition and Therapeutic Education, Department of Medicine, Geneva University Hospitals, Geneva, Switzerland

**Keywords:** Lipedema, Body composition, Dual-energy X-ray absorptiometry, Diagnostic test

## Abstract

**Introduction:**

Lipedema is a poorly known condition. Diagnosis is based almost exclusively on clinical criteria, which may be subjective and not always reliable. This study aimed to investigate regional body composition (BC) by dual-energy X-ray absorptiometry (DXA) in patients with lipedema and healthy controls and to determine cut-off values of fat mass (FM) indices to provide an additional tool for the diagnosis and staging of this condition.

**Methods:**

This study is a single-center case-control study performed at Lausanne University Hospital, Switzerland. Women with clinically diagnosed lipedema underwent regional BC assessment by DXA. The control group without clinical lipedema was matched for age and body mass index (BMI) at a ratio of 1:2 and underwent similar examination. Regional FM (legs, arms, legs and arms, trunk, android and gynoid FM) was measured in (kg) and divided by FM index (FMI) (kg/m<sup>2</sup>) and total FM (kg). The trunk/legs and android/gynoid ratios were calculated. For all indices of FM distribution showing a significant difference between cases and controls, we defined the receiver operating characteristic (ROC) curves, calculating the area under the curve (AUC), sensitivity, specificity, and Youden's index. Types and stages of lipedema were compared in terms of FM indices. Correlation analyses between all FM distribution indices and lipedema stages were performed.

**Results:**

We included 222 women (74 with lipedema and 148 controls). Overall, the mean age was 41 years (standard deviation [SD] 11), and mean BMI was 30.9 kg/m<sup>2</sup> (SD 7.6). A statistically significant difference was observed for all DXA-derived indices of FM distribution between groups, except for arm FM indices. The ROC curve analysis of leg FM/total FM, as a potential indicator of lipedema, resulted in an AUC of 0.90 (95% confidence interval 0.86–0.94). According to Youden's index, optimal cut-off value identifying lipedema was 0.384. Sensitivity and specificity were 0.95 and 0.73, respectively. We found no significant differences between lipedema types and stages in terms of FM indices, nor significant correlations between the latter and lipedema stages.

**Discussion/Conclusion:**

BC assessment by DXA, and particularly calculation of the leg FM/total FM index, is a simple tool that may help clinicians rule out lipedema in doubtful cases.

## Introduction

Lipedema is a chronic and progressive disease characterized by an abnormal deposition of subcutaneous adipose tissue leading to bilateral, disproportional enlargement of the extremities, typically sparing hands, feet, and trunk [1]. Lipedema can lead to considerable disability, impaired daily functioning, and psychosocial distress [2]. It affects almost exclusively women, starting between puberty and the third decade of life in most cases [3]. Although considered an orphan disease with an estimated prevalence of 1–9:100,000 in the general population [4], the exact prevalence of lipedema is still unknown and may be much higher than thought, reaching 39% of women according to a small German cross-sectional study [5]. In contrast to overweight and obesity, common weight loss strategies have no or limited influence on fat distribution in lipedema as these conditions do not share the same pathophysiology.

Diagnosis of lipedema is still based on clinical criteria proposed by Wold et al. [6]. One of the key criteria, the presence of a visual disproportion in body fat distribution, may be subjective and not always reliable between clinicians. As such, recognizing lipedema among conditions characterized by adiposity excess in lower limbs is particularly challenging. In the past, body mass index (BMI) has been suggested to help differentiate patients with and without lipedema [7]. However, BMI analysis may be misleading since it considers total weight without considering regional fat distribution, which is a hallmark of lipedema.

Several authors have attempted identifying parameters for detecting lipedema using radiological techniques, such as cutaneous ultrasonography [8, 9], magnetic resonance (MR) imaging [10], and non-contrast MR lymphography [11]. Recent research suggests that specific biomarkers [12], gene expression patterns [13, 14], and histological profiles [15] may also have a role in lipedema diagnosis in the near future. However, none of these tools have been yet validated or implemented in clinical practice.

Dual-energy X-ray absorptiometry (DXA) is a noninvasive, low-radiating imaging modality widely used to measure whole body bone mass and soft tissue composition [16, 17, 18, 19]. In recent years, DXA has become the reference tool in clinical routine to measure body composition (BC) and fat mass (FM) distribution [20, 21, 22, 23, 24]. In the setting of lipedema, two studies have evaluated possible BC differences in patients with lipedema compared to the general population using DXA [13, 25], and two others have applied DXA imaging to evaluate response to conservative treatment in patients with lipedema [26, 27]. In 2015, Dietzel et al. [25] reported the results of a study on 49 patients with lower limb lipedema and showed that the amount of leg FM divided by BMI was significantly higher among affected women as compared to 78 healthy subjects. The optimal cut-off value for leg FM/BMI identifying lipedema was 0.464 (kg/[kg/m^2^]), with an area under the curve (AUC) of 0.82 (95% confidence interval [CI] 0.75–0.90). Despite good sensitivity (0.87), diagnostic accuracy of this index seemed insufficient for clinical use because of low specificity (0.68). Furthermore, no information was provided in terms of correlation with type and stage of lipedema. Lastly, although BMI has been shown to correlate with DXA-derived FM with a correlation index (*r*) > 0.70 [28], it would be tempting to speculate that dividing regional FM by BMI might be misleading, as the latter index is dependent on the lean status of the subject. Lipedema is a disease concerning a priori only the adipose tissue distribution, and other indices based only on the FM might have a distinct advantage in this setting [16]. Given the potential of DXA to make disproportion in body fat distribution objective and measurable, the aims of our study were: (1) to investigate regional BC by DXA in patients with clinically diagnosed lipedema compared to healthy controls; (2) to determine appropriate cut-off values for meaningful DXA-derived indices of FM distribution capable of detecting lipedema and correlating with the different stages of the disease; and (3) to characterize DXA-based clinical phenotypes according to disease types and stages.

## Materials and Methods

### Population

A retrospective study was conducted in 74 adult female patients with clinically diagnosed lipedema who underwent BC assessment by DXA as part of their routine investigation at the Centre of Malformations and Rare Vascular Diseases of Lausanne University Hospital (Switzerland) between June 2018 and May 2020. Control group was represented by 148 women without clinical lipedema randomly enrolled from cross-sectional studies going on in the same institution, same period, and using the same DXA device (online Suppl. Materials and Methods; for all online suppl. material, see www.karger.com/doi/10.1159/000527138). Controls were group-matched at a ratio of 2 controls to 1 case for age (±3 years) and BMI (±3 kg/m^2^). All subjects were categorized into BMI ranges, according to the World Health Organization classification (normal weight: 18–24.9 kg/m^2^; overweight: 25–29.9 kg/m^2^; obesity class I: 30–34.9 kg/m^2^; obesity class II: 35–39.9 kg/m^2^; obesity class III: BMI ≥40 kg/m^2^) [29].

The study has been performed in accordance with the ethical standards of the 1964 Declaration of Helsinki and its later amendments, and the local ethics committee approved the study protocol (CER-VD, BASEC 2021-01265). All subjects gave their written informed consent for the use of their clinical data for research purposes.

### Lipedema Clinical Diagnosis

**Fig. 1:**
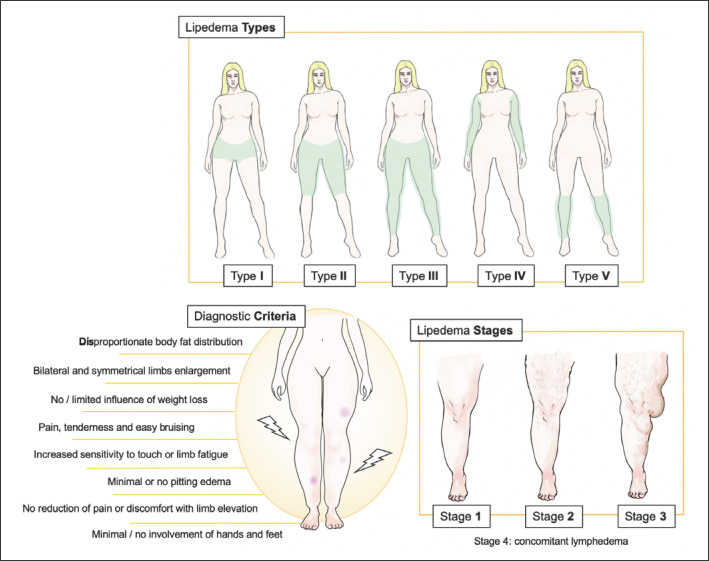
Recognized criteria for lipedema diagnosis and classification into types and stages. Type I: fat accumulation around the hips and buttocks. Type II: fat accumulation in the area from hips to knees. Type III: hip to ankle phenotype with a typical “cuff sign” at the ankle (i.e., fat deposits beginning abruptly above the malleoli). Type IV: fat accumulation in the arms (with or without lower limb involvement). Type V: fat dominating the calf region only. Stage 1: smooth and soft skin, underlying hypodermis thickened on palpation. Stage 2: skin indented over palpable pearl-sized nodules (“orange peel skin”). Stage 3: folds and divots over deforming, larger FMs. Stage 4: concomitant lymphedema.

Diagnosis of lipedema, as well as its classification into different types and stages, was made according to predefined clinical criteria [6, 30, 31], as outlined in Figure 1. In particular, diagnosis was retained when all the following clinical criteria were met: (i) disproportionate body fat distribution with bilateral and symmetrical enlargement of the limbs and minimal or no involvement of hands and feet; (ii) no or limited influence of weight loss on fat distribution; (iii) limb pain, tenderness, and easy bruising; (iv) increased sensitivity to touch or limb fatigue; (v) minimal or no pitting edema; (vi) no reduction of pain or discomfort with limb elevation. Lipedema type I was defined as fat accumulation around the hips and buttocks, type II as fat accumulation in the area from hips to knees, and type III as a hip to ankle phenotype with a typical “cuff sign” at the ankle (i.e., fat deposits beginning abruptly above the malleoli). Additional involvement of arms defined the type IV, while type V consisted of fat dominating the calf region only. In terms of severity, lipedema was classified as stage 1 if the skin was smooth and soft but the underlying hypodermis was thickened on palpation, stage 2 if the skin was indented over palpable pearl-sized nodules (“orange peel skin”), and stage 3 in case of folds and divots over deforming, larger FMs, while the development of concomitant lymphedema defined stage 4.

The Centre of Malformations and Rare Vascular Diseases of Lausanne University Hospital is a Swiss referral center for the management of lipedema, with more than 300 patients with confirmed diagnosis in regular follow-up. All individuals referred to the center for a suspicion of lipedema are evaluated by two angiologists with particular expertise in the setting of lipedema. Diagnosis and classification of the disease is confirmed in case of agreement between the two strictly following criteria listed above and in Figure 1. Difficult or otherwise uncertain cases are resolved during multidisciplinary meetings involving angiologists, plastic surgeons, and specialists in nuclear medicine.

### Anthropometric Measures

All participants had their height measured using the same portable stadiometer (Seca version 216, Seca) with a precision of 0.1 cm, and body weight was assessed with the same electronic scale (Seca Clara 803, Seca) with a precision of 0.1 kg, with the participant barefoot and in minimum clothing. BMI was then calculated by dividing the individual's weight by height squared (kg/m^2^).

### BC Assessment by DXA

BC assessment was performed using the Lunar iDXA® System (GE Medical Systems, Madison, WI, USA), in accordance with published guidelines by the International Society for Clinical Densitometry [32]. The scanners were calibrated daily using a standard calibration block supplied by the manufacturer. The GE Lunar iDXA® System has demonstrated excellent accuracy for BC assessments in individuals with normal weight and with obesity [33, 34].

All participants wore paper gowns and removed jewelry and any other personal items interfering with the DXA exam. Participants were placed in a supine position with palms down and arms at their sides, slightly separated from the trunk, and correctly centered on the scanning field. For patients that did not fit on the table, offset scanning method (mirror mode) of the overfilling member was used to avoid an underestimation of the measures [35]. Regions of interest were defined by the analytical program and included total body, trunk, head, pelvis, upper limbs, and lower limbs. First, automatic delimitation was used, then adjusted by the experienced human operator. For each region, DXA scanned weight of total mass, FM, and lean body mass. For the current study, relative BC measures of FM including legs, arms, legs and arms, trunk, android, and gynoid FM (kg) were derived by dividing them by FM index (FMI) (= regional FM/[total FM/height squared]) (kg/[kg/m^2^]) and by total FM (= regional FM/total FM) (kg/kg). Trunk/legs ratio was calculated as the ratio of FM in the trunk and FM in the legs. Android/gynoid ratio was calculated as the ratio of FM percentages in the android and gynoid regions.

The android region (area of the trunk between the ribs and the pelvis) is defined by the GE Lunar iDXA® System between the top of the iliac crest (lower boundary) and 20% of the distance between the pelvis and neck cuts (upper boundary). The upper boundary of the gynoid region (which includes hips and upper thighs, overlapping both leg and trunk regions) is set below the pelvis cut at 1.5 × android height, and gynoid height is determined as 2 × android region height [36].

Relative BC measures of lean mass (LM) including legs, arms, legs and arms, trunk, android and gynoid LM (kg) were derived by dividing them by LM index (LMI = LM/height^2^) (kg/m^2^) and by total LM (kg). The appendicular LM index (ALMI) was calculated as the ratio between leg and arm LM to height squared (kg/m^2^) and used both to define the LM distribution and as an indicator of sarcopenia [28, 37]. Total body bone mineral density (BMD) was calculated as total body bone mineral content divided by the bone surface from the total body scan excluding the head.

### Statistical Analysis

For comparison of quantitative variables between two groups of patients, the Student's *t* test or the Mann-Whitney test (in case of non-normal distribution) was performed. For comparison between three or more groups of patients, the one-way ANOVA test, the Kruskal-Wallis test (in case of non-normal distribution), and the post hoc Tuckey's test were used. Data are expressed as the mean ± standard deviation (SD).

For all DXA-derived indices of FM distribution showing a significant difference between patients with lipedema and controls, the diagnostic threshold for lipedema detection was defined based on receiver operating characteristic (ROC) curves, calculating AUC, sensitivity, specificity, and Youden's index (= sensitivity + specificity − 1).

Correlation analyses between indices of FM distribution and lipedema stages were performed with Pearson's or Spearman's test (in case of non-normal distribution). All the statistical tests were two tailed. Due to multiple comparisons (*n* = 27 indices tested), a Bonferroni-adjusted *p* value threshold of 0.00185 was applied. The analyses were performed with SPSS Statistics version 22.0 (IBM Corp., Armonk, NY, USA).

## Results

### Patients' General Characteristics

Seventy-four patients with lipedema were recruited by the Centre of Malformations and Rare Vascular Diseases of Lausanne University Hospital and 148 controls matched for BMI and age. The mean age was 40 years (SD 12) in cases and 41 years (SD 12) in controls. The mean BMI was also similar between groups: 31.3 kg/m^2^ (SD 8.9) in cases and 30.7 (SD 6.8) in controls. Following group matching, no statistically significant difference in terms of age, BMI, and FMI was found across all BMI ranges between cases and controls (shown in Table 1).

Among participants with lipedema, the disease was classified as type I in 3 patients (4%), type II in 11 patients (15%), type III in 29 patients (39%), type IV in 31 patients (42%), while no subject presented with type V lipedema. Disease severity was classified as stage 1 in 14 patients (19%), stage 2 in 39 patients (53%), stage 3 in 19 patients (26%), and stage 4 in 2 subjects (3%). Distribution of lipedema types and stages according to BMI ranges is shown in online Supplementary Table 1.

### DXA-Derived Indices of FM and LM Distribution

We first sought to study the difference between patients with lipedema and controls on FM and LM indices derived from regional assessment of BC. Overall, a statistically significant difference was found between cases and controls for all included indices of FM distribution except arm FM indices (shown in Table 2). Values were higher in cases for all limbs and gynoid-derived indices and lower for the trunk and android indices. When separately considering participants across BMI ranges, only the leg FM/total FM index consistently significantly differentiated cases and controls (see online Suppl. Table 2 for more details).

Concerning LM distribution, a statistically significant difference was found between patients with lipedema and controls only for leg LM/LMI, leg and arm LM/LMI, and leg LM/total LM, which were higher in cases, and arm LM/total LM, which was higher in controls (shown in Table 2). These differences did not remain significant when separately considering participants across BMI ranges (as shown in online Suppl. Table 3).

### Diagnostic Accuracy of Meaningful Indices of FM Distribution

**Fig. 2:**
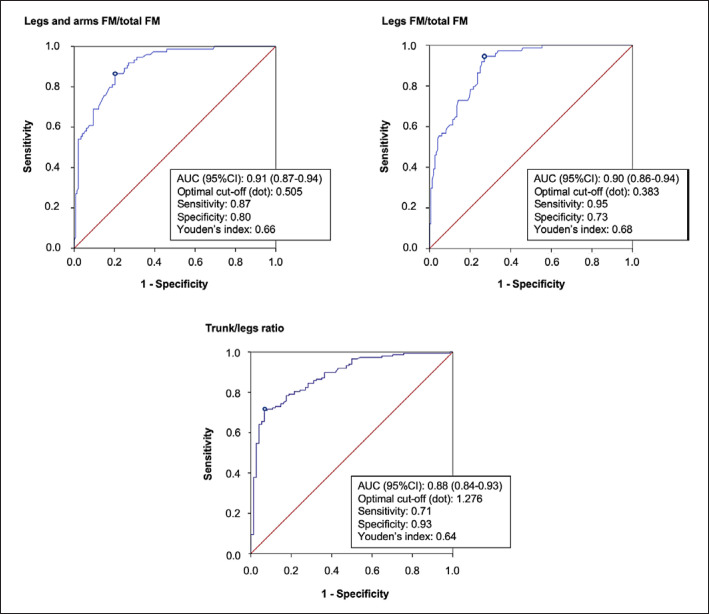
Area under the curve (AUC) and optimal cut-off value obtained from receiver operating characteristic (ROC) curve analysis of fat mass distribution indices with best diagnostic performance. AUC, area under the curve; BMI, body mass index; CI, confidence interval; FM, fat mass.

With regard to FM indices showing a significant difference between cases and controls, we performed ROC curve analyses to evaluate diagnostic accuracy and identify thresholds for lipedema detection. The indices of leg and arm FM/total FM, leg FM/total FM, and trunk/legs ratio provided the best diagnostic performances (shown in Fig. 2).

In particular, the leg and arm FM/total FM index displayed the best AUC (0.91; 95% CI: 0.87–0.94), the leg FM/total FM showed the best Youden's index (0.68) and sensitivity (0.95) for a cut-off of 0.383, whereas the trunk/legs ratio displayed the best specificity (0.93) for a cut-off of 1.276. Lower values of AUC were found for the other indices of FM distribution, as shown in online Supplementary Table 4.

### Indices of FM and LM Distribution and Bone Mineral Density in Patients with Lipedema according to Types and Stages

After exclusion of patients with lipedema type I due to low number (three subjects), no statistically significant difference was found between lipedema types with all indices of FM distribution, ALMI, and total body BMD (shown in Table 3 and in online Suppl. Table 5). After exclusion of lipedema stage 4 due to low number (two subjects), no statistically significant difference was found between lipedema stages with all indices of FM distribution, ALMI, and total body BMD (shown in Table 3 and in online Suppl. Table 5). Furthermore, no statistically significant correlation was found between lipedema stages and all indices of FM distribution (shown in Table 4).

## Discussion

In the present study, all measured DXA-derived indices of FM distribution, except for arm FM indices, were significantly different in patients with lipedema versus controls. Consistent with lipedema clinical characteristics, FM indices were higher in the lower part of the body (legs and gynoid region) in patients with lipedema, and those in the upper part (trunk and android region) in the controls. Among the former, leg and arm FM/total FM, leg FM/total FM, and trunk/legs ratio indices showed best diagnostic accuracy, with an AUC between 0.88 and 0.91, in detecting lipedema. The leg FM/total FM index displayed the best sensitivity (0.95) for a cut-off of 0.383 with a specificity of 0.73 using the Youden's index. Correlation analysis showed no statistically significant correlation between disease stages and all indices of FM distribution.

**Fig. 3:**
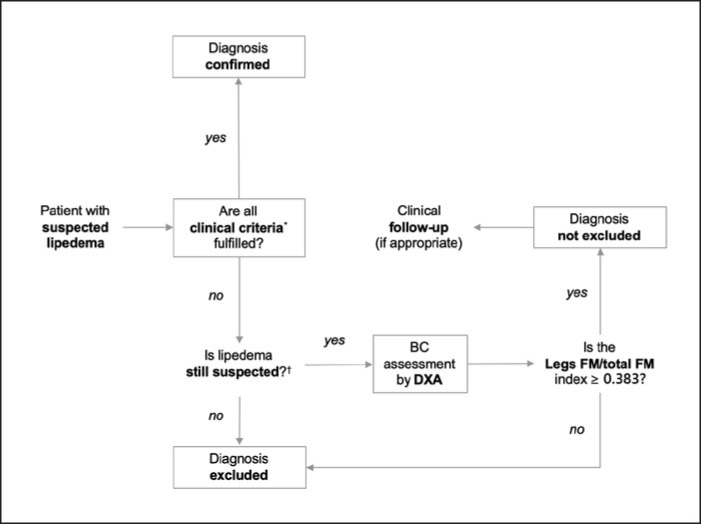
Proposed diagnostic algorithm in patients with suspected lipedema based on dual-energy X-ray absorptiometry (DXA)-derived leg FM/total FM index. DXA, dual-energy X-ray absorptiometry; FM, fat mass. *(i) Disproportionate body fat distribution with bilateral and symmetrical enlargement of the limbs and minimal or no involvement of hands and feet; (ii) no or limited influence of weight loss on fat distribution; (iii) limb pain, tenderness, and easy bruising; (iv) increased sensitivity to touch or limb fatigue; (v) minimal or no pitting edema; (vi) no reduction of pain or discomfort with limb elevation. ^†^Particularly in case of visual disproportion of body fat distribution questionable when other clinical criteria are fulfilled.

Compared with results published by Dietzel et al. [25], we found higher diagnostic accuracy for relative measures of FM distribution after adjustment for both FMI and total FM. Since lipedema is a disease concerning a priori only the adipose tissue distribution, it would be tempting to speculate that indices adjusted for FMI, which is independent of LM status, may have a distinct advantage over BMI in this context [16]. Interestingly, diagnostic efficacy was optimized in our study by dividing the amount of regional fat only by the total FM, thus removing height from the denominator. Both the leg FM/total FM and the leg and arm FM/total FM indices showed excellent AUC values. However, we believe that the former index has a particular advantage in this clinical setting, namely, the excellent sensitivity value. Since the diagnosis of lipedema is based on clinical criteria, the calculation of leg FM/total FM could be reserved for those cases that are doubtful according to standard criteria. Index values <0.383 would in that case allow to exclude the presence of disease with a reasonable degree of certainty. A possible diagnostic pathway in patients with suspected lipedema based on the leg FM/total FM index is shown in Figure 3.

Despite a statistically significant difference between cases and controls for some indices of LM distribution, overall, no such index maintained a significant difference in any of the BMI ranges in patients with lipedema compared with controls, including ALMI, which is currently considered a surrogate measure of skeletal muscle mass [38, 39]. This index also showed no significant differences among the different types and stages of lipedema. Similarly, total body BMD showed no significant differences between groups. Such results suggest that lipedema is a disease confined to adipose tissue, with no significant impact on the regional distribution of lean tissue or BMD.

Compared to MR imaging, DXA is a relatively simple and low-cost technique capable of investigating regional body fat distribution. Other BC analysis tools such as bioelectrical impedance analysis and plethysmography are burdened mainly by the fact that they cannot explore regional fat distribution [40]. Thus, DXA, as the gold standard for clinical routine analysis of regional BC analysis, could be the method of choice to make objective and measurable the disproportion in body fat distribution typical of lipedema. DXA could be particularly useful when such a disproportion is not visually striking, as in early stages or patients with obesity.

So far, few studies have investigated the diagnostic power of other techniques trying to identify diagnostic thresholds for the detection of lipedema. Quantification of platelet factor 4 levels in plasma exosomes in 15 patients with lipedema and 12 healthy controls resulted in an AUC of 0.95, with a sensitivity and specificity of 0.87 and 0.91, respectively, for a cut-off of 9.71 [12]. Another study on 89 women showed excellent performance of ultrasound in measuring the thickness of pretibial subcutaneous fat, with an AUC of 0.91 and a maximal sensitivity and specificity of 0.79 and 0.96, respectively, for a cut-off of 11.6 mm [41]. Lastly, using tissue dielectric constant measurement in 39 female patients, another study found a sensitivity and a specificity of 0.93 and 0.90, respectively, for a cut-off of 40 in differentiating women with untreated lower limb lymphedema from those with lipedema and healthy controls [42]. In the future, combining these and further techniques may facilitate lipedema diagnosis.

Our study has several limitations worth noting. Lipedema is a largely unknown and underdiagnosed disorder, and our sample size is relatively small. As the exact prevalence of the disease is currently unknown, it was not possible to include the calculation of the positive and negative predictive value of the various indices of FM distribution in the analysis of diagnostic accuracy. Moreover, types I and V were poorly represented in our cohort, whereas just 2 patients displayed lipedema stage 4. Based on our results, no conclusions may be drawn on these subsets of patients. Furthermore, there are no data in the literature regarding the ROC curves, sensitivity, and specificity of the clinical criteria used to diagnose lipedema, which have still been the gold standard for over 70 years. Lastly, although the vast majority of patients included in the different cohorts were of Caucasian origin, precise data on ethnicity are not available.

Despite these limitations, our study has considerable strengths. To our knowledge, this is the largest existing study involving BC assessment by DXA in patients suffering from lipedema, providing for the first time a quantifiable index of FM distribution with sufficient diagnostic accuracy for clinical use, independent of both BMI ranges and regional distribution of pathological fat (lipedema type).

We believe that such findings may pave the way for new research directions. First, our results should be validated in larger cohorts of patients with greater representation of all disease types. Future studies should particularly focus on patients with lipedema type IV, in whom FM distribution indices including arms may prove particularly useful. Moreover, research on populations with good representation of different ethnic groups should also investigate the influence of ethnicity on fat distribution in patients with lipedema. Notably, we found no significant correlation between all indices of FM distribution and lipedema stages after Bonferroni's adjustment, suggesting that such indices should not be used for disease staging. Longitudinal studies could rather evaluate their usefulness in monitoring disease evolution. In particular, such indices could serve as reliable and accurate tools in assessing clinical response to various standard or innovative therapies.

In the last 10 years, we have witnessed an increasing scientific interest in lipedema, reflecting a progressive awareness of the burden of this condition in the adult female population. Recognizing the disease in subjects with excessive adipose accumulation in the limbs is particularly important to avoid unnecessary and frustrating treatments, guide patients' expectations through education, and provide appropriate care [43]. Weight loss measures, including over-exercise, extreme dieting, weight loss treatments, or even bariatric surgery [44], exhibit minimal effect on the abnormal body fat distribution in patients with lipedema. These often unsuccessful interventions may even promote eating disorders, increase risk of depression, and other psychological disorders. The use of DXA in patients with suspected lipedema could allow appropriate early treatment and prevent late complications of the disease while also preventing affected patients from receiving inappropriate and even high-risk treatments such as bariatric surgery techniques [45].

To conclude, BC assessment by DXA, and particularly the leg FM/total FM index, might serve as a useful tool that may help clinicians in rule out lipedema. In light of the results of our study, we suggest implementing its use in clinical practice in combination with established clinical criteria. This index may be of particular use in patients with suspected clinical lipedema not fulfilling all the traditional diagnostic criteria. Our results also confirm that lipedema is a disease confined to adipose tissue, with no significant impact on the regional distribution of LM or total body BMD. Future research on larger populations is warranted to validate the results of our study.

## Statement of Ethics

Research has been performed in accordance with the ethical standards laid down in the 1964 Declaration of Helsinki and its later amendments, and local ethic committee (Commission cantonale d'éthique de la recherche sur l'être humain [CER-VD]) approved study protocol (Project-ID: 2021-01265). All subjects gave their written informed consent for the re-use of their clinical data for research purposes.

## Conflict of Interest Statement

The authors have no conflicts of interest to declare.

## Funding Sources

The authors received no specific funding for this work.

## Author Contributions

Giacomo Buso and Lucia Mazzolai contributed to the conception of the work. All authors contributed to the design of the work, as well as the acquisition and interpretation of data for the work. Giacomo Buso analyzed the data and drafted the manuscript. Lucie Favre, Nathalie Vionnet, Elena Gonzalez-Rodriguez, Didier Hans, Jardena Puder, Céline Dubath, Chin-Bin Eap, Wassim Raffoul, Tinh-Hai Collet, and Lucia Mazzolai critically revised the manuscript. All authors gave final approval and agree to be accountable for all aspects of the work, ensuring integrity and accuracy.

## Data Availability Statement

The data in this study were obtained from several cross-sectional studies (see also online suppl. materials and methods) where restrictions may apply. Such datasets may be requested from the principal investigators of the respective studies. Please contact Dr. Giacomo Buso (giacomo.buso@unil.ch) for further information.

## Figures and Tables

**Table 1:** Clinical characteristics in patients with lipedema compared with controls across all body mass index (BMI) ranges

Clinical characteristics	All BMI ranges			Normal weight (BMI 18–24.9 kg/m^2^)			Overweight (BMI 25–29.9 kg/m^2^)			Obesity class I (BMI 30–34.9 kg/m^2^)			Obesity class II (BMI 35–39.9 kg/m^2^)			Obesity class III (BMI ≥40 kg/m^2^)		
	overall	lipedema	controls	overall	lipedema	controls	overall	lipedema	controls	overall	lipedema	controls	overall	lipedema	controls	overall	lipedema	controls
Participants, *n* (%)	222	74 (33.3)	148 (66.7)	39	13 (33.3)	26 (66.7)	76	26 (34.2)	50 (65.8)	59	20 (33.9)	39 (66.1)	19	5 (26.3)	14 (73.7)	29	10 (34.5)	19 (65.5)
Age (years), mean (±SD)	41 (11)	40 (12)	41 (11)	34 (10)	31 (9)	36 (10)	41 (10)	40 (9)	41 (11)	43 (14)	42 (14)	44 (14)	43 (10)	45 (14)	42 (8)	41 (10)	45 (13)	39 (6)
BMI (kg/m^2^), mean (±SD)	30.9 (7.6)	31.3 (8.9)	30.7 (6.8)	22.7 (1.5)	23.2 (1.5)	22.4 (1.4)	27.3 (1.4)	27.2 (1.5)	27.4 (1.4)	31.9 (1.4)	32.0 (1.2)	31.9 (1.4)	36.2 (1.0)	35.7 (0.7)	36.4 (1.0)	45.8 (7.8)	49.1 (11.3)	44.1 (4.8)
FMI (kg/m^2^), mean (±SD)	12.71 (1.30)	13.03 (1.26)	12.43 (1.31)	7.53 (1.34)	8.06 (1.25)	7.26 (1.33)	11.06 (1.55)	11.27 (1.63)	10.95 (1.51)	14.30 (1.25)	14.86 (1.21)	14.00 (1.18)	17.27 (1.26)	16.99 (1.24)	17.37 (1.30)	22.76 (1.28)	23.63 (1.27)	22.53 (1.30)

Data are expressed as the mean±SD, regardless of the normality of the distribution. After Bonferroni's adjustment, Student's *t* test or the Mann-Whitney test (in case of non-normal distribution) showed no significant difference between patients with lipedema and controls across all BMI ranges. BMI, body mass index; FMI, fat mass index; SD, standard deviation.

**Table 2:** Dual-energy X-ray absorptiometry (DXA)-derived indices of fat (a) and lean (b) mass distribution in patients with lipedema compared with controls

(a) FM indices	Overall	Lipedema	Controls	*p* value
FM/FMI indices (kg/[kg/m^2^]), mean (±SD)				
Legs	1.039 (0.226)	1.237 (0.180)	0.940 (0.177)	**<0.001** ^a^
Arms	0.287 (0.043)	0.290 (0.048)	0.285 (0.040)	0.384^b^
Legs and arms	1.326 (0.236)	1.528 (0.189)	1.224 (0.187)	**<0.001** ^a^
Trunk	1.289 (0.209)	1.169 (0.229)	1.348 (0.171)	**<0.001** ^a^
Android	0.211 (0.050)	0.182 (0.044)	0.225 (0.047)	**<0.001** ^a^
Gynoid	0.491 (0.079)	0.532 (0.689)	0.469 (0.075)	**<0.001** ^b^
FM/total FM indices, mean (±SD)				
Legs	0.386 (0.070)	0.451 (0.050)	0.354 (0.055)	**<0.001** ^b^
Arms	0.107 (0.014)	0.106 (0.016)	0.107 (0.013)	0.183^a^
Legs and arms	0.493 (0.068)	0.556 (0.047)	0.462 (0.054)	**<0.001** ^b^
Trunk	0.482 (0.078)	0.427 (0.084)	0.510 (0.058)	**<0.001** ^a^
Android	0.079 (0.019)	0.066 (0.015)	0.085 (0.017)	**<0.001** ^b^
Gynoid	0.183 (0.023)	0.194 (0.018)	0.177 (0.023)	**<0.001** ^a^
Trunk/legs ratio, mean (±SD)	1.324 (0.444)	0.960 (0.252)	1.502 (0.411)	**<0.001** ^a^
Android/gynoid ratio, mean (±SD)	0.965 (0.176)	0.861 (0.155)	1.017 (0.164)	**<0.001** ^a^
(b) LM indices	Overall	Lipedema	Controls	*p* value
LM/LMI indices (kg/[kg/m^2^]), mean (±SD)				
Legs	0.977 (0.136)	1.039 (0.176)	0.946 (0.947)	**<0.001** ^b^
Arms	0.287 (0.040)	0.285 (0.054)	0.288 (0.031)	0.132^b^
Legs and arms	1.264 (0.165)	1.324 (0.222)	1.234 (0.118)	**<0.001** ^a^
Trunk	1.240 (0.149)	1.266 (0.209)	1.227 (0.106)	0.145^a^
Android	0.188 (0.039)	0.189 (0.032)	0.187 (0.020)	0.630^a^
Gynoid	0.413 (0.062)	0.424 (0.089)	0.407 (0.041)	0.013^a^
LM/total LM indices, mean (±SD)				
Legs	0.364 (0.035)	0.378 (0.052)	0.357 (0.018)	**<0.001** ^a^
Arms	0.107 (0.013)	0.104 (0.017)	0.109 (0.009)	**<0.001** ^a^
Legs and arms	0.471 (0.042)	0.482 (0.066)	0.466 (0.020)	0.005^a^
Trunk	0.463 (0.040)	0.461 (0.064)	0.463 (0.019)	0.004^a^
Android	0.070 (0.007)	0.069 (0.010)	0.070 (0.005)	0.003^a^
Gynoid	0.154 (0.018)	0.155 (0.029)	0.154 (0.009)	0.627^a^
ALMI (kg/m^2^), mean (±SD)	7.66 (1.31)	7.65 (1.62)	7.66 (1.12)	0.402^a^

Data are expressed as the mean±SD, regardless of the normality of the distribution. *p* values are in bold if statistically significant after Bonferroni's adjustment. ALMI, appendicular lean mass index; FM, fat mass; FMI, fat mass index; LM, lean mass; LMI, lean mass index; SD, standard deviation.

a The Mann-Whitney test was performed, as the distribution was non-normal.

b The Student's *t* test was performed, as the distribution was normal.

**Table 3:** Dual-energy X-ray absorptiometry (DXA)-derived indices of fat mass distribution according to lipedema types (a) and stages (b)

(a) FM indices	Overall (*N* = 74)	Type II (*N* = 11)	Type III (*N* = 29)	Type IV (*N* = 31)	*p* value
FM/FMI indices (kg/[kg/m^2^]), mean (±SD)					
Legs	1.237 (0.180)	1.210 (0.124)	1.291 (0.176)	1.184 (0.191)	0.059^a^
Arms	0.290 (0.048)	0.283 (0.050)	0.293 (0.057)	0.293 (0.040)	0.810^a^
Legs and arms	1.528 (0.189)	1.492 (0.146)	1.585 (0.174)	1.476 (0.210)	0.065^a^
Trunk	1.169 (0.229)	1.225 (0.208)	1.112 (0.156)	1.171 (0.168)	0.129^a^
Android	0.182 (0.044)	0.201 (0.038)	0.170 (0.043)	0.193 (0.140)	0.051^a^
Gynoid	0.532 (0.689)	0.557 (0.058)	0.543 (0.073)	0.510 (0.665)	0.082^a^
FM/total FM indices, mean (±SD)					
Legs	0.449 (0.050)	0.436 (0.047)	0.466 (0.051)	0.436 (0.047)	0.039^a^
Arms	0.106 (0.016)	0.101 (0.015)	0.106 (0.020)	0.108 (0.012)	0.529^a^
Legs and arms	0.555 (0.047)	0.537 (0.050)	0.557 (0.043)	0.544 (0.047)	0.023^a^
Trunk	0.419 (0.051)	0.439 (0.054)	0.401 (0.045)	0.433 (0.050)	0.110^a^
Android	0.067 (0.015)	0.072 (0.013)	0.061 (0.014)	0.071 (0.015)	0.011^a^
Gynoid	0.193 (0.018)	0.200 (0.017)	0.196 (0.019)	0.188 (0.016)	0.141^a^
Trunk/legs ratio, mean (±SD)	0.956 (0.219)	1.028 (0.225)	0.881 (0.193)	1.015 (0.225)	0.049^a^
Android/gynoid ratio, mean (±SD)	0.861 (0.155)	0.896 (0.140)	0.818 (0.163)	0.909 (0.144)	0.087^b^
(b) FM indices	Overall (*N* = 74)	Stage 1 (*N* = 14)	Stage 2 (*N* = 39)	Stage 3 (*N* = 19)	*p* value
FM/FMI indices (kg/[kg/m^2^]), mean (±SD)					
Legs	1.237 (0.180)	1.259 (0.159)	1.231 (0.183)	1.228 (0.194)	0.812^a^
Arms	0.290 (0.048)	0.297 (0.045)	0.294 (0.047)	0.273 (0.050)	0.232^a^
Legs and arms	1.528 (0.189)	1.558 (0.158)	1.525 (0.192)	1.504 (0.211)	0.655^a^
Trunk	1.169 (0.229)	1.082 (0.145)	1.144 (0.160)	1.254 (0.344)	0.076^b^
Android	0.182 (0.044)	0.157 (0.045)	0.181 (0.037)	0.199 (0.042)	0.036^a^
Gynoid	0.532 (0.689)	0.557 (0.048)	0.527 (0.072)	0.520 (0.071)	0.274^a^
FM/total FM indices, mean (±SD)					
Legs	0.452 (0.050)	0.464 (0.050)	0.449 (0.047)	0.449 (0.056)	0.595^b^
Arms	0.106 (0.016)	0.109 (0.013)	0.108 (0.018)	0.100 (0.015)	0.156^b^
Legs and arms	0.558 (0.047)	0.573 (0.043)	0.557 (0.046)	0.548 (0.053)	0.315^a^
Trunk	0.426 (0.085)	0.397 (0.045)	0.418 (0.048)	0.462 (0.141)	0.073^b^
Android	0.066 (0.015)	0.057 (0.015)	0.066 (0.013)	0.071 (0.016)	0.031^a^
Gynoid	0.194 (0.018)	0.205 (0.017)	0.192 (0.017)	0.190 (0.019)	0.044^b^
Trunk/legs ratio, mean (±SD)	0.960 (0.252)	0.874 (0.196)	0.952 (0.203)	1.052 (0.342)	0.202^b^
Android/gynoid ratio, mean (±SD)	0.862 (0.155)	0.760 (0.208)	0.863 (0.125)	0.929 (0.153)	0.009^b^

Data are expressed as the mean±SD, regardless of the normality of the distribution. *p* values are in bold if statistically significant after Bonferroni's adjustment. FM, fat mass; FMI, fat mass index; SD, standard deviation.

a The one-way ANOVA test was performed, as the distribution was normal.

b The Kruskall-Wallis test was performed, as the distribution was non-normal.

**Table 4:** Correlation analysis between indices of fat mass distribution and lipedema stages

FM indices	*r* value	*r*^2^ value	*p* value
FM/FMI indices			
Legs	–0.066	0.004	0.580^a^
Arms	–0.183	0.033	0.123^a^
Legs and arms	–0.109	0.012	0.360^a^
Trunk	0.239	0.057	0.043^b^
Android	0.295	0.087	0.012^a^
Gynoid	–0.172	0.030	0.148^a^
FM/total FM indices			
Legs	–0.096	0.009	0.422^b^
Arms	–0.217	0.047	0.067^b^
Legs and arms	–0.175	0.031	0.141^a^
Trunk	0.270	0.073	0.022^b^
Android	0.301	0.091	0.010^a^
Gynoid	–0.243	0.059	0.040^b^
Trunk/legs ratio	0.208	0.043	0.080^b^
Android/gynoid ratio	0.329	0.108	0.005^b^

FM, fat mass; FMI, fat mass index.

a The Pearson's test was performed, as the distribution was normal.

b The Spearman's test was performed, as the distribution was non-normal.
